# COVID-19 Lockdown in Patients with Chronic Diseases: A Cross-Sectional Study

**DOI:** 10.3390/ijerph19073957

**Published:** 2022-03-26

**Authors:** Mathieu Boulin, Amélie Cransac-Miet, Marc Maynadié, Fabienne Volot, Catherine Creuzot-Garcher, Jean-Christophe Eicher, Frédéric Chagué, Eléa Ksiazek, Guillaume Beltramo, Philippe Bonniaud, Thibault Moreau, Bernard Bonnotte, Edith Sales-Wuillemin, Agnès Soudry-Faure, Marianne Zeller, Yves Cottin

**Affiliations:** 1 Pharmacy Department, Dijon Bourgogne University Hospital, EPICAD LNC UMR 1231, 21000 Dijon, France; amelie.cransac@chu-dijon.fr; 2 Biological Haematology Department, Dijon Bourgogne University Hospital, Haematological Malignancies Registry, LNC UMR 1231, 21000 Dijon, France; marc.maynadie@u-bourgogne.fr; 3 Haemophilia Comprehensive Care Centre, Dijon Bourgogne University Hospital, 21000 Dijon, France; fabienne.volot@chu-dijon.fr; 4 Eye and Nutrition Research Group, Ophthalmology Department, Dijon Bourgogne University Hospital, UMR1324, INRAE, 6265 CNRS, 21000 Dijon, France; catherine.creuzot-garcher@chu-dijon.fr; 5 Cardiology Department, Dijon Bourgogne University Hospital, 21000 Dijon, France; jean-christophe.eicher@chu-dijon.fr (J.-C.E.); frederic.chague@chu-dijon.fr (F.C.); 6 Department of Clinical Research and Innovation (DRCI), Clinical Research Unit-Methodological Support Network (USMR), Dijon Bourgogne University Hospital, 21000 Dijon, France; elea.ksiazek@chu-dijon.fr (E.K.); agnes.soudry-faure@chu-dijon.fr (A.S.-F.); 7 Reference Constitutive Center for Rare Pulmonary Diseases, Pulmonary Medicine and Intensive Care Unit Department, Dijon Bourgogne University Hospital, LNC UMR 1231, 21000 Dijon, France; guillaume.beltramo@chu-dijon.fr (G.B.); philippe.bonniaud@chu-dijon.fr (P.B.); 8 Neurology Department, Dijon Bourgogne University Hospital, Bio-PeroxyIL, EA 7270, 21000 Dijon, France; thibault.moreau@chu-dijon.fr; 9 Department of Internal Medicine and Clinical Immunology, Dijon Bourgogne University Hospital, INSERM U1098, 21000 Dijon, France; bernard.bonnotte@chu-dijon.fr; 10 Psy-DREPI (Psychologie: Dynamiques Relationnelles et Processus Pdentitaires), University of Bourgogne Franche-Comté, EA 7458, 21000 Dijon, France; edith.sales-wuillemin@u-bourgogne.fr; 11 Cardiology Department, Dijon Bourgogne University Hospital, PEC2, EA 7460, 21000 Dijon, France; marianne.zeller@u-bourgogne.fr (M.Z.); yves.cottin@chu-dijon.fr (Y.C.)

**Keywords:** COVID-19, lockdown, chronic diseases, medication adherence, physician access, lifestyle behaviours, mental health, unhealthy behaviours

## Abstract

Background: We aimed to investigate the impact of the first COVID-19 lockdown on medication adherence, physician access, lifestyle behaviours, and mental health in patients with chronic conditions. Methods: A cross-sectional phone survey was conducted in 1274 housebound adults recruited from 8 regional chronic disease cohorts (CLEO CD study: NCT04390126). Results: Medication adherence was 97%; 305 (41%) patients declared that at least one scheduled visit with a physician was missed during the first lockdown. The main changes in lifestyle behaviours were deterioration in sleep time (duration and/or quality; 71%), increase in screen time (46%), and decrease in physical activity (46%). Nineteen percent experienced psychological distress (Kessler-6 score ≥ 5). An urban living place (OR, 1.76 vs. rural; 95% CI, 1.32–2.33; *p* = 10^−4^), worse self-reported mental health (OR, 1.62 vs. about the same or better; 95% CI, 1.17–2.25; *p* = 0.003), and a K6 score ≥ 5 (OR, 1.52 vs. <5; 95% CI, 1.05–2.21; *p* = 0.03) were independent factors associated with at least one unhealthy behaviour. Conclusions: Encouraging results were observed in terms of medication adherence. Caution is needed in chronic disease patients living in urban places as well as those presenting psychological distress and worse self-reported mental health to reduce unhealthy behaviours.

## 1. Introduction

On 11 March 2020, the World Health Organization characterized the novel severe acute respiratory syndrome coronavirus-2 (SARS-CoV-2) outbreak as a pandemic [1]. In an attempt to curtail the spread, the French government implemented a series of emergency measures on 17 March 2020, including the restriction of social contacts and the quarantine of coronavirus disease 2019 (COVID-19)-positive and suspected cases. Following the various policies implemented since the beginning of the pandemic, the potential consequences of COVID-19 lockdown and quarantine in patients with chronic diseases has become a major issue [2,3].

The French Restez Chez Vous decree [4] (meaning the “stay-at-home” decree) resulted in a sudden end radical change in the habits and lifestyles of the population. Physical distancing and self-isolation strongly impacted the general population, affecting eating habits and everyday behaviours in particular. In addition to the effect of the pandemic-related lockdown on individuals, health systems also switched their primary focus to pandemic containment and critical care management, which may have impaired scheduled and curative services, including emergency care in chronic patients.

There has been little investigation of the consequences of lockdown in patients with different chronic diseases [5]. We therefore conducted a cross-sectional study in order to explore the impact of the first COVID-19 lockdown on patient health indicators, including medication adherence, physician access, lifestyle behaviours, and mental health, among a cohort of housebound patients with chronic diseases located in Burgundy, which was at the centre of one of the first major COVID-19 outbreaks in France. Factors associated with unhealthy behaviours were also determined.

## 2. Materials and Methods

### 2.1. Design

The COVID-19-related Lockdown Effects on Chronic Diseases (CLEO-CD) study is a cross-sectional phone survey that was conducted among homebound patients with chronic conditions. Phone calls were made between 14 April 2020 and 2 June 2020 (i.e., at least 4 weeks after implementation of the French COVID-19 lockdown, which started on 17 March 2020). The CLEO-CD study was registered on ClinicalTrials.gov, accessed on 21 March 2022 (identifier: NCT04390126). Oral consent was obtained for each patient before enrolment.

### 2.2. Population

The CLEO-CD population includes patients with chronic diseases aged 18 years and over, living at home, and recruited from 8 regional (Burgundy, France) cohorts: Observatoire des Infarctus de Côte d’Or (RICO) for chronic coronary syndromes; Registre des Hemopathies Malignes de Côte d’Or (RHEMCO) for haematological malignancies; Haemophilia Comprehensive Care Centre for haemophilia; Reference Constitutive Centre for Rare Pulmonary Diseases for idiopathic pulmonary fibrosis and pulmonary arterial hypertension; age-related macular degeneration cohort; giant cell arteritis cohort; heart failure cohort; and multiple sclerosis cohort [6,7,8,9].

For rare diseases (giant cell arteritis, haemophilia, idiopathic pulmonary fibrosis, and pulmonary arterial hypertension), each cohort was enrolled in full. For other diseases, a sample was randomly drawn from each cohort or registry. A total of 1394 patients were contacted by phone for the study.

Exclusion criteria were not responding to one of three phone calls at different times of the day and different days of the week, inability to respond to a questionnaire (language barrier or cognitive impairment), refusal to participate, and death. In case of patients included in two or more cohorts, only data from the first phone call were kept for analysis. Patients were interviewed cohort by cohort, starting from patients with chronic coronary syndromes and finishing with haemophilia patients.

### 2.3. Survey and Questionnaires

Twelve trained interviewers collected data using standardised questionnaires by phone. The telephone interview lasted approximately 30 min. Patients responded to a general questionnaire and a specific questionnaire according to their cohort/chronic disease. Prior to the study, questionnaires were tested by a sample of 8 physicians (one per cohort) for content and 8 non-physicians (pharmacists or clinical research associates) for clarity. Minor changes were made before starting interviews.

In the general CLEO-CD questionnaire, patients were asked about socio-demographic data, including marital status, education level, living conditions (apartment, house, green space, or balcony), and place of residence (urban, >2000 inhabitants; rural, <2000 inhabitants) during lockdown. The questionnaire also assessed the impact of the COVID-19-related lockdown on overall physical and mental health, including psychological distress as well as lifestyle behaviours, including alcohol intake, smoking/vaping, eating and body weight, physical activity, screen time, and sleep quality and quantity. Psychological distress was assessed based on the Kessler Psychological Distress Scale K6 score [10]. The K6 scale is widely used and has proven reliability and validity across a wide variety of mental health surveys [10,11]. The scale includes six items associated with psychological distress during the previous 4 weeks. A K6 result of 5 or above indicates psychological distress [11]. Changes in lifestyle behaviours were reported as: increase, new behaviour, decrease, or no change.

Specific CLEO-CD questionnaires focused on disease-related symptoms/complications, management, and medication adherence. Patients were considered as non-adherent if they spaced out, discontinued, or changed the dosage of at least one of their current medicines without first consulting a healthcare professional (physician, nurse, pharmacist).

### 2.4. Statistical Analyses

Medication adherence, physician access, lifestyle behaviours, and mental health were described for each cohort and for the overall study population as frequencies and percentages for categorical data and means and standard deviations for continuous data. Logistic regression models were used to identify factors associated with at least one unhealthy patient behaviour, including medication non-adherence, increase in alcohol intake/new drinker, increase in cigarette consumption/new smoker, increase in vaping/new user, decrease in physical activity, deterioration in sleep time or quality, increase in screen time, and increase in body weight. Variables that reached a significance of *p* < 0.20 in univariate analyses were introduced into a multiple logistic regression analysis. Highly correlated variables were not used together in the logistic regression models in order to avoid collinearity. During the backward elimination process, variables were excluded if the corresponding *p*-value for the Wald test was higher than 0.05. All statistical analyses were performed using SAS statistical software (SAS Institute, Cary, NC, USA).

## 3. Results

### 3.1. Baseline Patient Characteristics

A total of 1274 patients participated in the study. Of the 1394 eligible patients, 120 (9%) participants were excluded either for not responding to the three phone calls (*n* = 86, 6%), death (*n* = 22, 2%), inability to respond (*n* = 10, 1%), refusal to participate (*n* = 9, 1%), or hospitalization (*n* = 1, <1%). Two (<1%) patients from the haematological malignancy cohort were not interviewed because they had already been interviewed in the heart failure or multiple sclerosis cohort.

Mean age was 65.5 ± 16.8 years, and 55% of patients were men. At the time of the phone call, 63 (5%) patients had been tested for SARS-CoV-2 by polymerase chain reaction, and only one was found to be positive. Other baseline characteristics are reported in Table 1 for each chronic disease cohort.

### 3.2. Impact of COVID-19-Related Lockdown

#### 3.2.1. Medication Adherence and Physician Access

In the full cohort, 33 (3%) patients declared that they discontinued and/or modified the dosage of at least one of their current medicines themselves without any medical advice. The proportion of medication non-adherence varied from 0% (giant cell arteritis and heart failure cohorts) to 11% (multiple sclerosis cohort). The main reason given to explain the lack of adherence was fear (fear of contracting COVID-19 while visiting the hospital for intravitreal injections, fear of developing a more severe form of COVID-19 with some of their medicines especially immunosuppressive agents, nonsteroidal anti-inflammatory drugs or angiotensin-converting enzyme inhibitors, etc.). Media influence was reported by only two patients to explain non-adherence. Among the 738 (58%) patients that reported having one or more scheduled visits with their general practitioner or another physician during the period, 305 (41%) declared that they did not attend at least one visit. The frequency of missed scheduled visits was the highest in the chronic respiratory diseases cohort (39%), while the proportion of performed scheduled visits (phone call, telehealth, or physical) was the highest in the haematological malignancy group (57%) (Table 2).

#### 3.2.2. Lifestyle and Mental Health

Lifestyle and mental health changes compared to the pre COVID-19 period are detailed in Table 2 for each cohort. Overall, the main changes in lifestyle behaviours were deterioration in sleep time (duration and/or quality; 71%), increase in screen time (46%), and decrease in physical activity (46%). The multiple sclerosis cohort had the lowest rate of deterioration in sleep time, the highest rate of increase in screen time, and the highest rate of decrease in physical activity. Minor differences were observed among cohorts for these three behaviours. Alcohol intake was slightly modified (increase, 5%; decrease, 10%), and increases in cigarette consumption/new smokers or increases in vaping/new users were rare, with frequencies of 4% and 1%, respectively. An increase in body weight was reported in approximately one-fourth of the patients, varying from 14% of the age-related macular degeneration cohort to 32% of the haemophilia cohort. Almost 30% of patients self-reported worse mental health, and 19% had a K6 score higher or equal to five. Only minor differences between cohorts were observed in terms of mental health or alcohol, cigarette, or vaping habits.

### 3.3. Factors Associated with at Least One Unhealthy Behaviour

Eight hundred and forty-nine (69%) patients reported at least one unhealthy behaviour. One, two, three, and at least four unhealthy behaviours were reported in 453 (37%), 328 (27%), 64 (5%), and 4 (0.5%) patients, respectively. Among the 453 patients with one unhealthy behaviour, the most frequent were an increase in screen time (*n* = 217, 48%) and a decrease in physical activity (*n* = 208, 46%). Among the 328 patients with two unhealthy behaviours, the most frequent behaviours were (equally frequent) increase in screen time and decrease in physical activity (*n* = 281, 86%).

In univariate analysis, factors associated with at least one unhealthy behaviour were gender, age, education level, marital status, living place, residence type, nature of the chronic disease, history of depression, self-reported mental health, and K6 score. In the final logistic regression model, three factors were independently associated with at least one unhealthy behaviour: an urban living place (OR, 1.76 vs. rural; 95% CI, 1.32–2.33; *p* = 10^−4^), worse self-reported mental health (OR, 1.62 vs. about the same or better; 95% CI, 1.17–2.25; *p* = 0.003), and a K6 score ≥ 5 (OR, 1.52 vs. below 5; 95% CI, 1.05–2.21; *p* = 0.03) (Table 3).

## 4. Discussion

In a large, community-dwelling population of 1274 participants with one or several chronic diseases, we found that during the first lockdown, medication adherence remained high (97%). In contrast, we reported a deterioration in lifestyle behaviours, in particular sleep time, screen time, and physical activity.

Our study has major strengths. The CLEO-CD population was composed of patients with chronic diseases aged 18 years and over, living at home, and recruited from eight regional (Burgundy, France) cohorts. The rate of participation (91%) compares favourably with most health-related surveys, highlighting the high level of patient implication for this subject. Most studies or reviews that reported COVID-19-related consequences included patients with chronic diseases of the same type, mostly auto-immune/inflammatory [12], neurologic [13], oncologic [14], or cardiovascular [15]. The reason is probably hypotheses about the risk of infection or severity of COVID-19 associated with specific types of drugs (immunosuppressive and anticancer agents, corticosteroids and nonsteroidal anti-inflammatory drugs, angiotensin-converting enzyme (ACE) inhibitors, and angiotensin receptor blockers), which has been highlighted since the beginning of the pandemic, including in the medical literature [16]. Logically, we chose to include chronic patients exposed to one of these medication classes in our analysis but also other chronic patients exposed to drugs that were not thought to influence COVID-19 infection or severity, for example, haemostatic agents in haemophilia patients or antiangiogenics in age-related macular degeneration patients. Second, to be as representative as possible of the general population of chronic disease patients, we included all of the patients from our regional cohorts for rare diseases, such as giant cell arteritis, or samples randomly assigned from registries for more frequent diseases, such as chronic coronary syndromes. Finally, the major strength of our study is that it provides new findings regarding the consequences of the COVID-19-related lockdown for multiple health indicators, including mental health and lifestyle.

We found encouraging results for adherence to medication in our population (97% overall). Not surprisingly, the lowest levels of adherence were observed in multiple sclerosis patients treated with immunosuppressive agents because of fear of developing a life-threatening form of COVID-19 and in age-related macular degeneration patients who refused to go to hospital for intravitreal injections because of fear of contamination. ACE inhibitors were also discontinued in patients with chronic coronary syndromes or heart failure based on initial speculations that the use of ACE inhibitors, leading to increased expression of ACE2, which could potentially facilitate infection with COVID-19. Our observation highlights the ability of the patients and/or their family members to effectively self-manage and their discernment regarding alarming information circulating in the media during the first lockdown. Only 2 of 1274 patients declared that the media influenced non-adherence, and none blamed their loss of adherence on limited healthcare access. This positive result may be explained by the rapid onset of regional management protocols in accordance with the French or international recommendations, such as those established by the World Federation of Haemophilia for optimal management and follow-up of haemophilia patients [17]. For instance, the strict application of patient supply protocols between the hospital pharmacy, haemophilia comprehensive care centre, and patients ensured that there was no shortage of haemostatic treatments. Telehealth was rapidly implemented for some chronic diseases and probably explains the low percentage of postponed visits in patients with a haematological malignancy during the period. A major limit for the development of telehealth, in particular in low- and middle-income countries, is the lack of technology, tools, and investment. These factors likely explain the deleterious COVID-19-related consequences described in chronic Indian patients [18]. We also reported a moderate rate of psychological distress (19%) among our 1274 patients. This is probably explained by the elevated rate of patients living in rural areas (55%) and with an outdoor space (76%).

In terms of lifestyle behaviours, our results were globally in line with the literature in healthy patients. Surveys conducted between March and June 2020, in which participants reported their perceived changes in lifestyle factors since the start of the pandemic, underscored a general worsening of sleep quality [19,20], mild or no changes in cigarette and alcohol consumption [19,21], slight or no changes in body weight [21,22], conflicting results for diet quality [23,24], considerable reductions in physical activity, and increases in sedentary time [19,25,26]. Reassuringly, the proportion of alcohol drinkers who decreased their consumption in our study was approximately two-fold higher than that of those who increased their consumption (10% vs. 5%), which may be due to the more social nature of drinking in Southern European countries [21,27] as frequently declared by our patients during interviews. Conversely, approximately one-fourth of our population said that they had gained body weight. Changes in body weight occurring in short time periods may be alarming if, as the literature on obesity suggests, they remain over time [28]. We collected data on body weight, which we found to be more relevant than data on eating habits.

Unlike most of the reviewed web-based surveys, the CLEO-CD study relied on telephone interviews and used information on participants’ health behaviours and health status collected in-home before the pandemic, thus reducing the risk of information bias (i.e., people not accurately reporting their past exposure or symptoms). As the COVID-19 epidemic evolves, and new social distancing measures are implemented, and in case of future pandemics, it is critical to identify population subgroups that have an increased risk of deteriorated health. This is particularly important for older patients because they live more frequently alone and are most vulnerable to the development and progression of diseases, including COVID-19, in the absence of medical follow-up. To our knowledge, our study is the first to identify urban living place, psychological distress, and worse self-reported mental health as independent factors associated with at least one unhealthy behaviour in individuals with chronic diseases.

Our study has four main limitations. First, it was restricted to a single French region, so our results cannot be generalized. Second, our patients had mainly rare, chronic diseases. They were not representative to all chronic patients. The third limitation is the absence of patient follow-up. Lastly, due to the design of our study, we did not compare our lifestyle-related findings with those reported in healthy individuals in other studies.

## 5. Conclusions

To conclude, we found a very high rate of medication adherence (97%) during the first COVID-19-related lockdown in a French region that was an early epicentre of the pandemic in Europe. This encouraging result may partly be explained by the adequation of previously established protocols. In contrast, we reported a deterioration in lifestyle behaviours, in particular sleep time, screen time, and physical activity. Among patients with chronic conditions, those living in an urban environment as well as those presenting psychological distress or mental health issues need to be carefully monitored to reduce unhealthy behaviours. The adequacy of previously established protocols may explain.

## Figures and Tables

**Table 1:** Baseline patient characteristics.

	Overall Population (*n* = 1274)	Haematological Malignancy (*n* = 252)	Chronic Respiratory Diseases (*n* = 123)	Giant Cell Arteritis (*n* = 74)	Age-Related Macular Degeneration (*n* = 200)	Chronic Coronary Syndrome (*n* = 219)	Multiple Sclerosis (*n* = 100)	Heart Failure (*n* = 123)	Haemophilia (*n* = 183)
	N (%)	N (%)	N (%)	N (%)	N (%)	N (%)	N (%)	N (%)	N (%)
Gender									
Female	572 (45)	129 (51)	45 (37)	56 (76)	108 (54)	65 (30)	83 (83)	49 (40)	37 (20)
Male	702 (55)	123 (49)	78 (63)	18 (24)	92 (46)	154 (70)	17 (17)	74 (60)	146 (80)
Age, years (mean ± SD)	65.5 ± 16.8	67.9 ± 13.9	70.2 ± 11.0	77.2 ± 7.5	77.7 ± 9.7	66.4 ± 12.0	46.3 ± 13.0	70.0 ± 13.9	47.6 ± 18.1
Education level									
Middle school	349 (27)	56 (22)	37 (30)	16 (22)	47 (24)	70 (32)	20 (20)	39 (32)	64 (35)
High school	429 (33)	92 (37)	49 (40)	34 (46)	96 (48)	65 (30)	11 (11)	47 (38)	35 (19)
Graduation and above	455 (36)	101 (40)	34 (28)	18 (24)	48 (24)	73 (33)	66 (66)	33 (27)	82 (45)
Marital status									
Married	821 (64)	176 (70)	79 (64)	47 (64)	113 (57)	154 (70)	72 (72)	71 (58)	109 (60)
Divorced	102 (8)	23 (9)	11 (9)	7 (9)	14 (7)	15 (7)	11 (11)	11 (9)	10 (5)
Widowed	190 (15)	30 (12)	18 (15)	18 (24)	67 (34)	25 (11)	2 (2)	23 (19)	7 (4)
Single	135 (11)	20 (8)	13 (11)	1 (1)	6 (3)	17 (8)	15 (15)	18 (15)	45 (25)
Place of residence during lockdown									
Rural (<2000 inhabitants)	696 (55)	132 (52)	77 (63)	27 (37)	115 (58)	111 (51)	52 (52)	68 (55)	114 (62)
Urban (≥2000 inhabitants)	562 (44)	110 (44)	46 (37)	46 (62)	83 (42)	106 (48)	47 (47)	55 (45)	69 (38)
Residence type during lockdown									
House with garden	866 (66)	169 (67)	83 (67)	49 (66)	144 (72)	142 (65)	67 (67)	77 (63)	135 (74)
House without garden	33 (3)	9 (4)	2 (2)		8 (4)	6 (3)	1 (1)	2 (2)	5 (3)
Apartment with outdoor	239 (19)	54 (21)	26 (21)	18 (24)	32 (16)	38 (17)	16 (16)	28 (23)	27 (15)
Apartment without outdoor	127 (10)	17 (7)	12 (10)	6 (8)	14 (7)	31 (14)	15 (15)	16 (13)	16 (9)
Job status									
Not working before and during lockdown	998 (78)	213 (85)	117 (95)	71 (96)	191 (96)	166 (76)	45 (45)	107 (87)	88 (48)
Part-time working	58 (5)	3 (1)			3 (2)	14 (6)	8 (8)	5 (4)	25 (14)
Working at home	63 (5)	8 (3)	2 (2)		1 (1)	12 (5)	18 (18)	2 (2)	20 (11)
History of depression									
No	987 (78)	198 (79)	92 (75)	58 (78)	156 (78)	176 (80)	70 (70)	108 (88)	129 (70)
Yes	281 (22)	54 (21)	30 (24)	16 (22)	44 (22)	39 (18)	30 (30)	15 (12)	53 (29)

**Table 2:** Patient impact of COVID-19-related lockdown.

	Overall Population (*n* = 1274)	Haematological Malignancy (*n* = 252)	Chronic Respiratory Diseases (*n* = 123)	Giant Cell Arteritis (*n* = 74)	Age-Related Macular Degeneration (*n* = 200)	Chronic Coronary Syndrome (*n* = 219)	Multiple Sclerosis (*n* = 100)	Heart Failure (*n* = 123)	Haemophilia (*n* = 183)
	N (%)	N (%)	N (%)	N (%)	N (%)	N (%)	N (%)	N (%)	N (%)
**Medication non-adherence**	33 (2.6)	1 (0.4)	1 (0.8)		10 (5.0)	2 (0.9)	11 (11.0)		8 (4.4)
**Scheduled medical visit(s)**									
None	500 (40.4)	50 (19.8)	36 (29.3)	32 (43.2)	73 (36.5)	112 (51.1)	34 (34.0)	55 (44.7)	108 (59.0)
Not performed (1 or more)	305 (24.6)	54 (21.4)	48 (39.0)	21 (28.4)	34 (17.0)	50 (22.8)	25 (25.0)	33 (26.8)	40 (21.9)
Performed (including telehealth)	433 (35.0)	143 (56.8)	36 (29.3)	21 (28.4)	83 (41.5)	46 (21.0)	40 (40.0)	29 (23.6)	35 (19.1)
**Lifestyle**									
Alcohol intake									
No/no change	1086 (85.2)	217 (86.1)	95 (77.2)	66 (89.2)	184 (92.0)	192 (87.7)	89 (89.0)	108 (87.8)	135 (73.8)
Increase	64 (5.0)	10 (4.0)	7 (5.7)	5 (6.8)	4 (2.0)	10 (4.6)	4 (4.0)	5 (4.1)	19 (10.4)
Decrease	124 (9.7)	25 (9.9)	21 (17.1)	3 (4.1)	12 (6.0)	17 (7.8)	7 (7.0)	10 (8.1)	29 (15.9)
Cigarette consumption									
No/no change	1204 (94.6)	248 (98.4)	117 (98.5)	73 (98.7)	189 (94.5)	200 (91.3)	93 (93.0)	119 (96.8)	161 (88.0)
New smoker	9 (0.7)	1 (0.4)			1 (0.5)	2 (0.9)	2 (2.0)	1 (0.8)	2 (1.1)
Increase	37 (2.9)	1 (0.4)		1 (1.4)	7 (3.5)	8 (3.7)	4 (4.0)	2 (1.6)	14 (7.7)
Decrease	22 (1.7)	2 (0.8)	2 (1.6)		3 (1.5)	8 (3.7)	1 (1.0)	1 (0.8)	5 (2.7)
Vaping									
No/no change	1243 (98.7)	250 (99.2)	120 (97.6)	74 (100)	200 (100)	209 (95.4)	97 (97)	121 (98.4)	171 (92.6)
New user	2 (0.2)								2 (1.1)
Increase	6 (0.5)	1 (0.5)				1 (0.5)			4 (2.2)
Decrease	9 (0.7)		1 (0.8)				3 (3.0)		5 (2.7)
Physical activity									
No change or increase	682 (54.2)	133 (52.8)	54 (43.9)	37 (50.0)	116 (58.0)	120 (54.8)	44 (44.0)	73 (59.4)	105 (57.4)
Decrease	576 (45.8)	116 (46.0)	60 (48.8)	37 (50.0)	83 (41.5)	96 (43.8)	56 (56.0)	50 (40.6)	78 (42.6)
Sleep time/quality									
Increase (one or both)	358 (28.6)	82 (32.5)	33 (26.8)	18 (24.3)	40 (20.0)	52 (23.7)	43 (43.0)	31 (25.2)	59 (32.2)
Decrease (one or both)	894 (71.4)	167 (66.3)	87 (70.7)	53 (71.6)	155 (77.5)	163 (74.4)	57 (57.0)	88 (71.5)	124 (67.8)
Screen time increase	587 (46.3)	123 (48.8)	57 (46.3)	31 (41.9)	72 (36.0)	98 (44.8)	60 (60.0)	56 (45.5)	90 (49.2)
Body weight change									
No change	727 (60.9)	145 (57.5)	74 (60.2)	44 (59.5)	132 (66.0)	134 (61.2)	43 (43.0)	63 (51.2)	92 (50.3)
Increase	290 (24.3)	58 (23.0)	24 (19.5)	12 (16.2)	28 (14.0)	52 (23.7)	23 (23.0)	34 (27.6)	59 (32.2)
Decrease	176 (14.8)	36 (14.3)	16 (13.0)	12 (16.2)	17 (8.5)	26 (11.9)	23 (23.0)	19 (15.5)	27 (14.8)
Mean ± SD	0.14 ± 2.48	−0.11 ± 3.06	0.39 ± 2.93	0.00 ± 1.68	0.08 ± 1.40	0.13 ± 1.72	−0.24 ± 1.64	0.59 ± 3.48	0.25 ± 2.49
**Mental health**									
Self-reported mental health									
About the same or better	884 (69.4)	171 (67.9)	88 (71.5)	52 (70.3)	137 (68.5)	167 (76.3)	51 (51.0)	98 (79.7)	120 (65.6)
Worse	380 (29.8)	77 (30.6)	35 (28.5)	22 (29.7)	60 (30.0)	50 (22.8)	48 (48.0)	25 (20.3)	63 (34.4)
K6 score (mean ± SD) *	2.6 ± 3.3	3.0 ± 3.2	2.0 ± 3.4		1.9 ± 3.0	3.3 ± 4.0	2.9 ± 3.4	2.3 ± 3.0	2.4 ± 3.0
K6 score (class) *									
<5	933 (73.2)	179 (71.0)	97 (78.9)		170 (85.0)	155 (70.8)	78 (78.0)	100 (81.3)	154 (84.2)
≥5	242 (19.0)	66 (26.2)	19 (15.5)		28 (14.0)	57 (26.0)	20 (20.0)	23 (18.7)	29 (15.9)

K6, Kessler Psychological Distress Scale (K6 scale). * No data were collected for patients with giant cell arteritis.

**Table 3:** Factors associated with at least one unhealthy behaviour: multivariate analysis.

Factor		Odds Ratio	95% CI	*p*-Value
Gender	Female vs. male	1.16	0.85; 1.58	0.34
Age		0.99	0.98; 1.00	0.07
Marital status				0.87
	Single vs. widowed	1.00	0.53; 1.89	
	Divorced vs. widowed	0.90	0.48; 1.69	
	Married vs. widowed	0.87	0.56; 1.35	
Education level				0.12
	High school vs. graduation and above	0.82	0.58; 1.16	
	Middle school vs. graduation and above	0.72	0.52; 1.01	
Place of residence during lockdown	Urban vs. rural	1.76	1.32; 2.33	**10^−4^**
Residence type during lockdown	House/apartment without outdoor vs. with outdoor	1.29	0.77; 2.17	0.34
Chronic disease *				0.69
	Chronic respiratory diseases vs. multiple sclerosis	1.23	0.59; 2.58	
	Age-related macular degeneration vs. multiple sclerosis	0.80	0.40; 1.60	
	Chronic coronary syndrome vs. multiple sclerosis	0.84	0.43; 1.62	
	Heart failure vs. multiple sclerosis	0.77	0.38; 1.57	
	Haemophilia vs. multiple sclerosis	0.83	0.43; 1.61	
	Haematological malignancy vs. multiple sclerosis	0.77	0.41; 1.47	
History of depression	Yes vs. no	1.13	0.81; 1.59	0.48
Self-reported mental health	Worse vs. about the same or better	1.62	1.17; 2.25	**0.003**
K6 score (class)	≥5 vs. <5	1.52	1.05; 2.21	**0.03**

95% CI, 95% confidence interval; K6, Kessler Psychological Distress Scale (K6 scale). * No data for giant cell arteritis because of missing data for K6 score.

## Data Availability

The data presented in this study are available on request from the corresponding author. The data are not publicly available due to University Hospital of Dijon property rules.

## References

[1] World Health Organization. https://www.who.int/emergencies/diseases/novel-coronavirus-2019.

[2] Mauro V., Lorenzo M., Paolo C., Sergio H. (2020). Treat all COVID 19-positive patients, but do not forget those negative with chronic diseases. Intern. Emerg. Med..

[3] Mansfield K.E., Mathur R., Tazare J., Henderson A.D., Mulick A.R., Carreira H., Matthews A.A., Bidulka P., Gayle A., Forbes H. (2021). Indirect acute effects of the COVID-19 pandemic on physical and mental health in the UK: A population-based study. Lancet Digit. Health.

[4] Journal Officiel de la République Française Décret n° 2020-293 du 23 mars 2020 Prescrivant les Mesures Générales Nécessaires Pour Faire Face à L’épidémie de COVID-19 Dans le Cadre de L’état D’urgence Sanitaire. https://www.legifrance.gouv.fr/loda/id/LEGIARTI000041759981/2020-03-27/.

[5] Flint S.W., Brown A., Tahrani A.A., Piotrkowicz A., Joseph A.C. (2020). Cross-sectional analysis to explore the awareness, attitudes and actions of UK adults at high risk of severe illness from COVID-19. BMJ Open.

[6] Cransac-Miet A., Zeller M., Chagué F., Soudry-Faure A.S., Bichat F., Danchin N., Boulin M., Cottin Y. (2021). Impact of COVID-19 lockdown on lifestyle adherence in stay-at-home patients with chronic coronary syndromes: Towards a time bomb. Intern. J. Cardiol..

[7] Praliaud R., Greigert H., Samson M., Zeller M., Boulin M., Bielefeld P., Ramon A., Cottin Y., Bonnotte B. (2021). Impact of the COVID-19 lockdown on the management and control of patients with GCA. Ann. Rheum. Dis..

[8] Chagué F., Boulin M., Eicher J.C., Bichat F., Jalmes M.S., Cransac-Miet A., Soudry-Faure A., Danchin N., Cottin Y., Zeller M. (2020). Impact of lockdown on patients with congestive heart failure during the coronavirus disease pandemic. ESC Heart Fail..

[9] Beltramo G., Cransac A., Favrolt N., Spanjaard M., Zeller M., Cottin Y., Boulin M., Bonniaud P. (2021). Impact of the COVID-19 lockdown suufering from idiopathic interstitial pneumonia. Respir Med. Res..

[10] Kessler R.C., Andrews G., Colpe L.J., Hiripi E., Mroczek D.K., Normand S.-L.T., Walters E.E., Zaslavsky A.M. (2002). Short screening scales to monitor population prevalences and trends in non-specific psychological distress. Psychol. Med..

[11] Schulz R., Beach S.R. (1999). Caregiving as a risk factor for mortality: The Caregiver Health Effects Study. JAMA.

[12] Picchianti Diamanti A., Cattaruzza M.S., Di Rosa R., Porto F.D., Salemi S., Sorgi M.L., Martin L.S.M., Rai A., Iacono D., Sesti G. (2020). Psychological Distress in Patients with Autoimmune Arthritis during the COVID-19 Induced Lockdown in Italy. Microorganisms.

[13] Piano C., Di Stasio E., Primiano G., Janiri D., Luigetti M., Frisullo G., Vollono1 C., Lucchini M., Brunetti V., Monforte M. (2020). An Italian Neurology Outpatient Clinic Facing SARS-CoV-2 Pandemic: Data From 2167 Patients. Front. Neurol..

[14] Erdem D., Karaman I. (2020). Awareness and perceptions related to COVID-19 among cancer patients: A survey in oncology department. Eur. J. Cancer Care Engl..

[15] Aajal A., El Boussaadani B., Hara L., Benajiba C., Boukouk O., Benali M., Ouadfel O., Bendoudouch H., Zergoune N., Alkattan D. (2021). The consequences of the lockdown on cardiovascular diseases. Ann. Cardiol. Angeiol..

[16] Bavishi C., Maddox T.M., Messerli F.H. (2020). Coronavirus Disease 2019 (COVID-19) Infection and Renin Angiotensin System Blockers. JAMA Cardiol..

[17] World Federation of Haemophilia COVID-19 (Coronavirus Disease 2019) Pandemic Caused by SARS-CoV-2: Practical Recommendations for People with Hemophilia. https://news.wfh.org/covid-19-coronavirus-disease-2019-pandemic-caused-by-sars-cov-2-practical-recommendations-for-hemophilia-patients/.

[18] Gautam V., Dileepan S., Rustagi N., Mittal A., Patel M., Shafi S., Thirunavukkarasu P., Raghav P. (2021). Health literacy, preventive COVID-19 behaviour and adherence to chronic disease treatment during lockdown among patients registered at primary health facility in urban Jodhpur, Rajasthan. Diabetes Metab. Syndr..

[19] Balanzá-Martínez V., Kapczinski F., de Azevedo Cardoso T., Atienza-Carbonell B., Rosa A.R., Mota J.C., De Boni R.B. (2021). The assessment of lifestyle changes during the COVID-19 pandemic using a multidimensional scale. Rev. De Psiquiatr. Y Salud Ment. (Engl. Ed.).

[20] Robillard R., Dion K., Pennestri M.H., Solomonova E., Lee E., Saad M., Murkar A., Godbout R., Edwards J.D., Quilty L. (2021). Profiles of sleep changes during the COVID-19 pandemic: Demographic, behavioural and psychological factors. J. Sleep. Res..

[21] López-Moreno M., López M.T.I., Miguel M., Garcés-Rimón M. (2020). Physical and Psychological Effects Related to Food Habits and Lifestyle Changes Derived from Covid-19 Home Confinement in the Spanish Population. Nutrients.

[22] Fernandez-Rio J., Cecchini J.A., Mendez-Gimenez A., Carriedo A. (2020). Weight changes during the COVID-19 home confinement. Effects on psychosocial variables. Obes. Res. Clin. Pract..

[23] Rodríguez-Pérez C., Molina-Montes E., Verardo V., Artacho R., García-Villanova B., Guerra-Hernández E.J., Ruíz-López M.D. (2020). Changes in Dietary Behaviours during the COVID-19 Outbreak Confinement in the Spanish COVIDiet Study. Nutrients.

[24] Marty L., de Lauzon-Guillain B., Labesse M., Nicklaus S. (2021). Food choice motives and the nutritional quality of diet during the COVID-19 lockdown in France. Appetite.

[25] Castañeda-Babarro A., Arbillaga-Etxarri A., Gutiérrez-Santamaría B., Coca A. (2020). Physical Activity Change during COVID-19 Confinement. Int. J. Environ. Res. Public Health.

[26] Stanton R., To Q.G., Khalesi S., Williams S.L., Alley S.J., Thwaite T.L., Fenning A.S., Vandelanotte C. (2020). Depression, Anxiety and Stress during COVID-19: Associations with Changes in Physical Activity, Sleep, Tobacco and Alcohol Use in Australian Adults. Int. J. Environ. Res. Public Health.

[27] Scarmozzino F., Visioli F. (2020). Covid-19 and the Subsequent Lockdown Modified Dietary Habits of Almost Half the Population in an Italian Sample. Foods.

[28] Bhutani S., Cooper J.A. (2020). COVID-19-Related Home Confinement in Adults: Weight Gain Risks and Opportunities. Obesity Silver Spring.

